# Constructing the *school paradox* in the lives of children living with parental mental illness

**DOI:** 10.1177/13591045231154112

**Published:** 2023-01-23

**Authors:** Ebenezer Cudjoe, Cherry HL Tam, Marcus YL Chiu

**Affiliations:** 1 Centre for Childhood Studies, Department of Psychosocial and Psychoanalytic Studies, 2591 University of Essex, UK; 2 Department of Social and Behavioural Sciences, 53025 City University of Hong Kong, Hong Kong; 3 School of Health & Wellbeing, 1796 University of Bolton, UK; Centre for Mental Health and Society, Bangor University, Wales, UK

**Keywords:** Children, parental mental illness, parent, school, phenomenology

## Abstract

Children living with parental mental illness are referred to as an invisible population because mental health services rarely target them, as the focus is often on the parent who is ill mentally. The same situation occurs even in school where they are unnoticed. This study conducted in Ghana creates awareness about what these children think about their interactions at school in the context of parental mental illness. Data was collected through interviews and diaries with 13 children living with parental mental illness and analysed to attain the essential features through Husserl’s transcendental phenomenology. The children find the school as a happy space where they do not have to be worried about the parent’s mental illness. Ultimately, though, even at school, most of the children become concerned about the mental wellbeing of the parent due to their loyalty towards them. This results in the school paradox where the children are torn between having their own time at school and being worried about the parent’s condition back home, wanting to be there for the parent. The school paradox is an unhealthy cycle that could be addressed with coordinated efforts from mental health professionals, social workers, psychologists and teachers.

## Introduction

Research indicates that there is an estimation of 20% of children who have parents with mental illness across the general population (Maybery et al., 2009). Parental mental illness can affect children in several ways. For those with severe mental illness, hallucinations, delusions and depression could occur which can negatively interfere with how they respond to the needs of their children (Campbell & Poon, 2020). It is also not uncommon to find parents with mental illness lose custody of their children due to neglect (Hollingsworth, 2004), which can subsequently lead to poor outcomes for the child. One of the settings that the children frequent is the school. However, it is unclear how these children navigate interactions in the school setting while living with a parent with mental illness. The children’s interactions at school refers to their day-to-day activities with peers, teachers, classroom work, break time activities and play. Understanding how children perceive the school, as someone whose parent has mental illness, could help conceptualise interventions and supports that clarify where professionals can focus their efforts. This study explored what children living with a parent with mental illness think about their interactions at school.

During symptomatic phase of their mental illness, parents can be abusive or neglectful (Cudjoe, 2022; Strand et al., 2020). However, the same parent can demonstrate warmth and affection to the child when they present no symptoms. This can make the child feel anxious when around the parent. Therefore, going to school may be considered a welcome escape from the chaos they may encounter at home. School can provide these children with a space where they may not have to think or be worried about their parent’s condition because they can find opportunities for diversion (Gladstone et al., 2011; Hosman et al., 2009). However, the school can also be a place of additional stress due to the potential stigma that these children can receive from their peers such as bullying. Thus, while the school may offer children some hope to focus on their own life and enjoy that moment, there is also the risks for negative experiences. A kind of cycle is created as the children move from home to school due to their association with parental mental illness. The cycle is what we call the “school paradox”. The school paradox is unfolded in the discussion section, backed by evidence from our empirical data.

The stigma surrounding mental illness can also make it difficult for children to talk about mental illness in school (Reupert et al., 2021). This is more so in Ghana, where this study was conducted, with the pervasive stigma and the lack of adequate mental health services to reach these families. The children may not be willing to speak with professionals when there is inadequate attention to mental health issues in Ghana (Bird et al., 2011). At the school, teachers have a responsibility to identify and meet the needs of these children. However, it is usually the case that these teachers do not consider themselves as having the skills to help the children (Laletas et al., 2017; Reupert & Maybery, 2007). In fact, some teachers may not even be aware of the child who has a parent with mental illness in their school. Considering that the school is a place that children frequent the most, it is important that professionals like school psychologists, social workers and teachers are aware of the child’s family situation and understand how it impacts their school involvement. However, such coordinated efforts from professionals are rare due to the limited understanding of the children’s situation at school.

There is a lack of early identification of children living with parental mental illness in schools which also leads to the unavailability of coordinated efforts among professionals to meet the needs of these children (Laletas et al., 2020). Indeed, while teachers have expressed the wish to support these children, they also acknowledge that they are ill-prepared in terms of their skills and knowledge (Bibou-Nakou, 2004; Laletas et al., 2017). A part of the lack of coordinated support for these children is that there is inadequate understanding into what they make of their school interactions. A better understanding of how these children navigate interactions at school could be used as a guide to suggest areas where professionals can focus their efforts to promote positive outcomes for the children.

## Method

### Study Design and Recruitment

This study followed concepts from Husserl’s transcendental phenomenology, particularly that of the natural attitude and inter-subjectivity. The natural attitude encapsulates our involvement in a world that is already meaningful which leads to our tendency to take things for granted (Luft, 1998). We are rarely reflective or critical of our ordinary day-to-day activities. But to be phenomenological, we need to question some of the taking-for-grantedness. Inter-subjectivity represents the exploration of a phenomenon in a way that is shared by a community with a similar experience. These concepts enable to reach the essential features of a phenomenon which was the main goal of the approach. This study investigates the phenomenon of “school setting” for a child who has a parent with mental illness. It unfolds what goes on in the school for these children.

The study focused on collecting data from children ages 10 to 17 who live with a parent with mental illness. Recruitment of participants for the study was done by contacting two out-patient mental health units in Ghana for information on patients receiving services who were also parents. It was a challenge to access such information because the mental health professionals did not collect information on the parental status of their patients. However, the professionals assisted by contacting their patients and finding out if they had children from 10 to 17 years. Together, the out-patient units provided a list of 17 families with a parental mental illness with a child from ages 10 to 17. Of the 17 families, 14 agreed to have their children participate in the study. After contacting the families, 13 children participated in the interviews. One child did not give assent even though the parent had consented to have them involved in the study.

### Participants

Children, aged 10 to 17, living with a parent with mental illness participated in the study. Of the 13 children, five were males and eight females. The children were either in basic (*n* = 6) or secondary school (*n* = 7). All the children came from families that were separated and most of the time lived with the parent with mental illness. Their parents’ diagnosis included psychosis, schizophrenia, depression and anxiety disorders. Generally, these diagnoses represent conditions that involve changes in emotion, thinking and/or behaviour (American Psychiatric Association, 2020).

### Data Collection: Phenomenological Interviews and Diaries

The interviews were guided by ideas from transcendental phenomenology. The beginning of the interviews focused on the naïve experiences as we are often absorbed into a pre-existing world (Bevan, 2014). At this stage, questions were asked to attain descriptive responses. For example, the children were asked to describe a regular day at school. Following this, the next stage of questioning was more analytical to apprehend the phenomenon. Also, because a goal of the approach was to reach the essential features of the phenomenon, imaginative variation was used where imaginative questions were asked to strip the phenomenon of its accidental properties (Zahavi, 2018). For example, the children were asked to think about how their life would have been if they spent all their days at school. All interviews, which lasted from 40 to 60 minutes, were conducted at the participants’ homes.

The children were also given 21 days to complete a diary as part of the data collection procedure. The diary was made with A4 papers containing empty spaces where the children were expected to write about things they thought about in terms of living with a parent with mental illness. This study reports on what the children reflected on from their engagement with school. The first page of the diary included some guidelines to help them complete the diary. For example, they were informed that they could draw or write in the diary, write as clearly as possible and not worry about spelling mistakes and not to fill in the diary for a day that they forgot. The children were reminded once a week so they did not forget to make entries. Of the 13 children who were interviewed, eight agreed to complete the diary. A complete description of the diary including the diary completion guideline can be found in Cudjoe (2022).

### Data Analysis

The goal of data analysis was to get to the essence of the phenomenon in terms of what children living with parental mental illness make of their interactions at the school setting. The start of data analysis was to read through all interview transcripts for familiarisation. After this, stories were developed for each interview to better understand the lifeworld of the participants. Questions were then asked of the transcribed data and stories to develop meaning units where the focus was on the meaning in the data (Dahlberg et al., 2008). The meaning units were then clustered to form the essential themes presented in the results section. In order to reveal the essential features that define the phenomenon and makes it what it is, the interview data was depersonalised (Larsen & Adu, 2022). With this, the analysis did not involve demographic features of participants as is often done in idiographic analysis. This was because the focus of analysis in transcendental phenomenology is on the phenomenon, not the person.

### Ethics

This study was approved by the Ethics Committee of City University of Hong Kong. Parents/guardians were asked to give informed consent before the children were involved in the study. All children also had to provide their assent showing that they were participating in the study out of their volition. Thus, although parents/guardians provided consent, final decision rested on the child participant. The children’s role in the study as participants was clearly explained to them before they had to agree. For instance, they were made to understand that they could be interviewed for about 60 minutes, that they were not obligated to answer every question, agreed to have the interviews audio-recorded and could request their interview to be deleted. The children also understood that participation in the study was not connected with any provision of social and mental health services. A mental health nurse accompanied the interviewer throughout the process and was available to provide immediate counselling support or connect the child to relevant services if they felt any discomfort during the interview.

## Results

### Essence of the Phenomenon

The results are presented through a structure of meanings. The first is to present the essential meanings before its constituents. The essential meaning gives the key features of the phenomenon that makes it what it is while the constituents give further details into particular features and nuances of the phenomenon. The results are mostly presented in the present tense as it shows the essential features that makes the phenomenon what it is.

The essence of the phenomenon “school setting” for a child living with parental mental illness comprises a space for self-reflection, being away from parental mental illness, being in the moment and enjoying that moment. Meanwhile the self-reflection can often be interrupted by thoughts about the parent with mental illness. So, while the child is alone at school and is expecting to focus on activities there, their thoughts sometimes drift to that of worry or concern towards the parent’s condition. More so, the self-reflection and opportunities that the school can give these children seem to rely on them keeping information about their parent’s mental illness a secret.

The following constituents of the phenomenon presented here relates to how the children navigate interactions at the school setting. The themes: (i) school as a happy space, (ii) keeping school in the dark and (iii) Impact on school setting, define what the children think about their interactions at school. Table 1 below shows a general overview of the constituents as extracted from NVivo. The “references” column refers to how often the participants referred to the topic.

**Table 1.** Description of constituents extracted from NVivo 12 (edited).

Cluster of meanings (constituents)	Meaning units’ description	Number of participants	References
School as a happy space	The school is one place that the children don’t have to think about their parent’s mental illness. Just to learn and have fun	10	41
Keeping school in the dark	The school is not informed about the parent’s mental illness. Child thinks they can’t help. They don’t want others to know	13	70
Impact on school setting	Child is unable to concentrate in school because they worried/think about the parent’s condition	8	39

### School as a Happy Space

School as a happy space represents a phenomenon of the school as a place where it is just about school, not being worried or reflecting on having a parent with mental illness. The children see the school as a place for learning, playing or having fun, while for some it is just the feeling of moving away from the parent with mental illness that makes them happy. These children liked to talk about routine activities at school which they seem to enjoy.

In the morning when you arrive at school, you first sweep and then you read your book. When your madam comes then she starts teaching (Abigail)

School is fine, I like it there because everything is okay. There is time for everything, you always know what to do and I like it (Ajoa)

**Figure 1.**
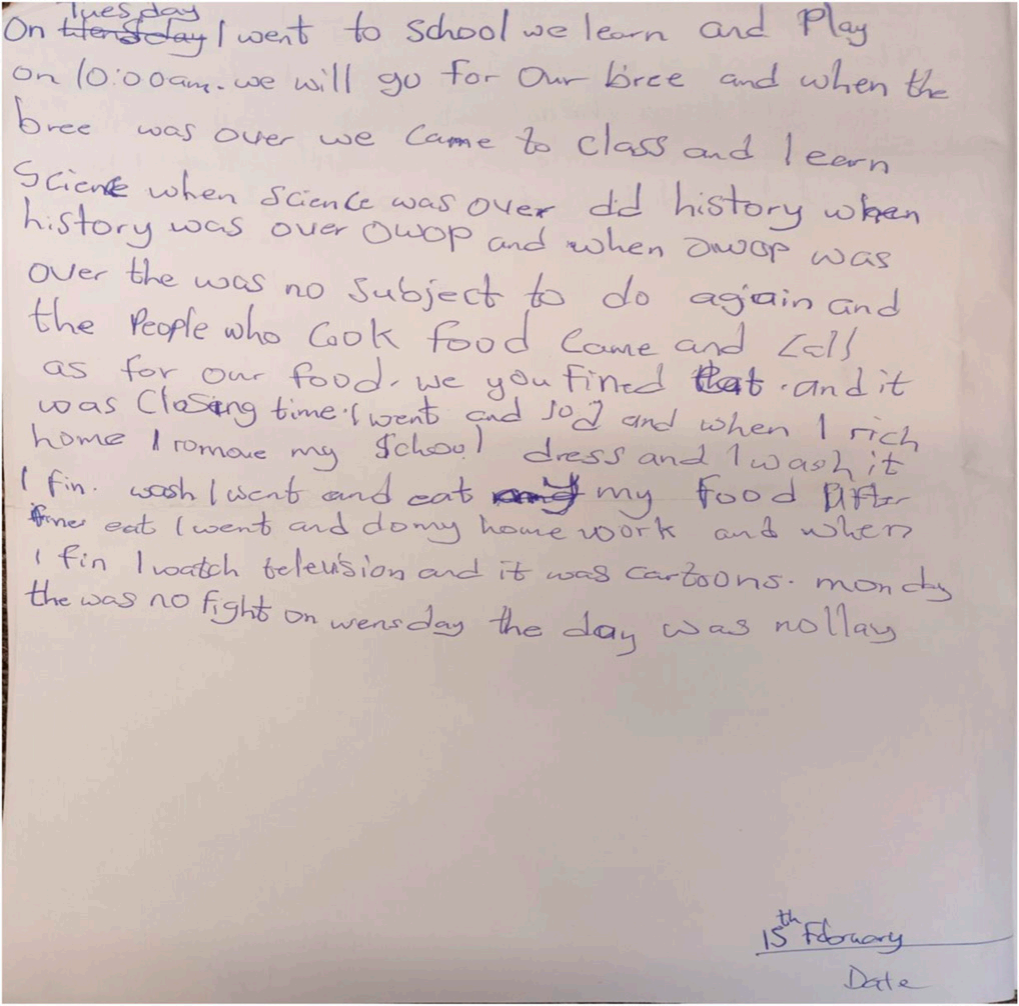
Routine activities at school (Rosemary).

The school provides an order of activities for the children which many of them mention were not used to at home. Figure 1 below is an excerpt from one child’s diary which further shows how these children are accustomed to routine activities that they expect to be involved in at school.

The school is also a happy space because it is a setting where children encounter many peers of similar ages they can play and have fun with. The children can engage in fun activities with peers that makes them not focused on their parent’s mental illness. It is not uncommon that children living with a parent with mental illness can be burdened with thoughts of their parent’s illness. The burden can be about who gives them medication, how the parent is doing at home especially if they are often unstable, and thoughts about who takes the parent to hospital when their symptoms show. However, once children immerse themselves in school activities, it is possible that their mind is removed from these thoughts. Therefore, it is not always intentional that children want to not think about their parent. But the spontaneous flow of activities at school and how it make them happy overshadows thoughts about happenings at home. It can also be deliberate efforts by the child as they actively use the school as an opportunity to keep their distance from home or the parent.

I prefer the school. There are friends there who will make me happy because if I stay home there are no friends (Christabel)

I enjoy school a lot. There are teachers I am friendly with, they help me learn. At home even if I say something my mother will not listen to me. I like it here *[at school]* (Emma)

Comparing school to home, the children see many happy moments there than at home living with a parent with mental illness. This is understandable as their family can often be characterised by strenuous relationships. Therefore, their transition into the school setting is to take advantage of the personal space they enjoy: “It is much better in school than home. When I get home he *[parent with mental illness]* has been insulting people” (Kojo).

### Keeping School in the Dark

A common theme that appears in the lifeworld of the children is that, at the school setting, they do not want people to know about their parents’ mental illness. They believed that they should keep information about their parents’ mental illness to themselves. Indeed, many of these children worried that they might not enjoy their time at school if peers or, even for some, teachers, got to know about the mental illness. There is, in fact, evidence that children are likely to be stigmatised by their peers once they are aware that the friend has a parent with mental illness (Gladstone et al., 2011). The school is a “happy space” and they do not want the pattern to be disturbed due to sharing information about parental mental illness with others.

No, I don’t want it to spread because some of the students they will think that your mother is mad (Ben)

Even if I tell them *[peers and teachers]* they will not help me. Some of them don’t really care (Joyce)

The children often do not want to give it a chance by informing the school about parental mental illness. It is possible that this is being influenced by family and social factors. There are many instances that parents inform children not to talk to or tell others about mental illness in the family. As loyal as they are to their parents, children oblige not to reveal such family secret. This is one way their ability to open up about mental illness at school is being impacted by family expectations. On the other hand, social factors, particularly regarding stigma and discrimination that children perceive, also seem to impact whether or not they talk to people at school about their parent’s mental illness.

Due to the parents’ mental illness, especially those with severe conditions, they may not be actively involved in the child’s school activities. It is possible that teachers may question the child about their parent’s non-involvement in school activities like the PTA. However, they have no intention to talk about their parent. Some of the children understand that it is not the parent’s fault that they miss out on being present for their child at school.

Oh, I’m not proud that my mother doesn’t know about what goes on at school. But it’s not her fault, it’s due to her illness (Christabel)

**Figure 2.**
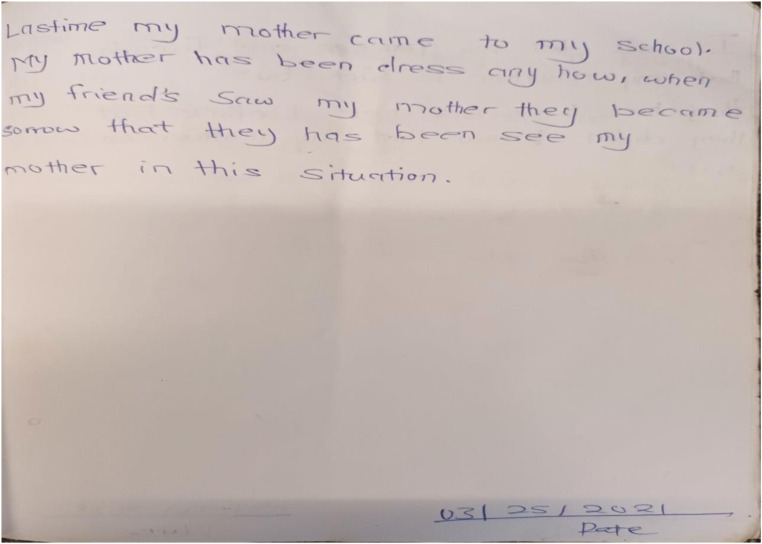
Not happy to see parent in poor mental state at school (Naomi).

The presentation from the diary below (Figure 2) is an illustration of the point that some children may not be happy for their peers or teachers to see the parent in their state of poor mental health.

The children do not want other people to see their parents under the situation where they show their symptoms as it makes them uncomfortable. There is the assumption that other people at school do not share similar experience with them, so, it is important that they keep the information out of school.

It’s not good for my mother to be here *[school]* in that condition, people don’t understand and they react anyhow. For the PTA I don’t give it to my mother. I think that when she goes maybe she will shout and people may say she is mentally ill. That is why I have not been informing her about school activities (Rosemary)

### Impact on School Setting

The children also described various ways having a parent with mental illness can impact their life at school. The outcome is usually negative as it makes them uncomfortable in school. For many children they are unable to focus in classroom work because they either have to leave the class and attend to their ill parent at home or they will be in the classroom but their continuous thoughts of how well their parents are doing back home distorts their involvement in classroom work. Some of the children also bring to school a sense of worry, especially when they have immediately had a disturbing engagement with their parent on the way to school. Therefore, the transition from home to the school setting is not always smooth for these children.

It is really worrying. It *[having a parent with mental illness]* affects my education because I don’t get the peace of mind to study because every day I think about it (Emma)

Even sometimes when I go to school and they are teaching I don’t feel fine (Christabel)

The emotional or psychological troubles the children go through at home is transferred to the school setting. Essentially, while the school brings physical distance between the child and parent with mental illness, it does not necessarily relief them of emotional burden. Physical distance does not guarantee a clear emotional space. On the first thought, the child may think that being in school gives them the personal space to reflect on their own lives without having to think about their parent’s mental illness. However, this is not simple especially if the child is most impacted by the parent’s mental illness while at home.

For these children, navigating the complexities of school in the context of parental mental illness means putting their academic performance and involvement in other school-related activities at risk. In the classroom setting, children see themselves as not being able to concentrate on what is being taught. The disruption to their concentration is because they also have to think about their parent who is ill. There is therefore a competition for space in their heads between what they are being taught in class and what they are thinking about their parent’s mental illness. The inability to process these thoughts clearly can result in poor academic performance due to lack of concentration.

At first when the illness was serious I used to come home and sleep due to that my academics wasn’t good. I was doing okay but since daddy got ill my grades have been dropping. Sometimes I cry. Sometimes when I am in school they *[parent without mental illness]* can call that daddy has been admitted to the hospital and I have to leave class and go (Ogla)

**Figure 3.**
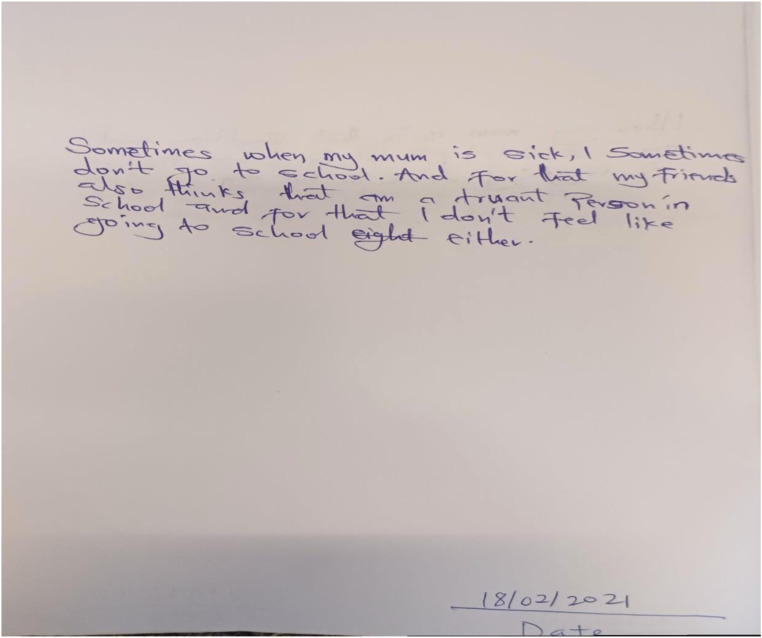
Staying away from school due to parent’s mental illness (Christabel).

As seen from the quote, for some children, they may have to leave school, or skip a class, to check on their parent or provide some form of support. The excerpt from the diary below (see Figure 3) shows that, sometimes, the child also has to stay home because of the parents’ condition. This can be more problematic for those children who take on most caregiving responsibilities.

## Discussion

The study has revealed the children’s life situation within the school setting when living with parental mental illness. Previous research has shown that children can experience challenging circumstances like abuse and neglect while living with a parent with mental illness (Cudjoe et al., 2022; Hollingsworth, 2004). Therefore, it is important that these children have a space for self-reflection where they focus on themselves. This study shows that the school is one setting that can provide children with the space to focus on their own lives and have some happy moments as well. However, the idea of the school as a happy space can be messy once they enter the school. The children do not tell the school about the parent’s mental illness and many of them are negatively impacted as they are unable to concentrate in class and their grades could also fall because of caring responsibilities. In line with this, it is not surprising that research indicated teachers often do not seem qualified or prepared to meet the needs of these children (Laletas et al., 2017; Reupert & Maybery, 2007). While studies have explored how professionals like teachers can support these children, there is limited understanding about the mechanism within the school that creates problematic outcomes for children. This study reveals the mechanism creating poor outcomes for the children in school.

### Developing the School Paradox

**Figure 4.**
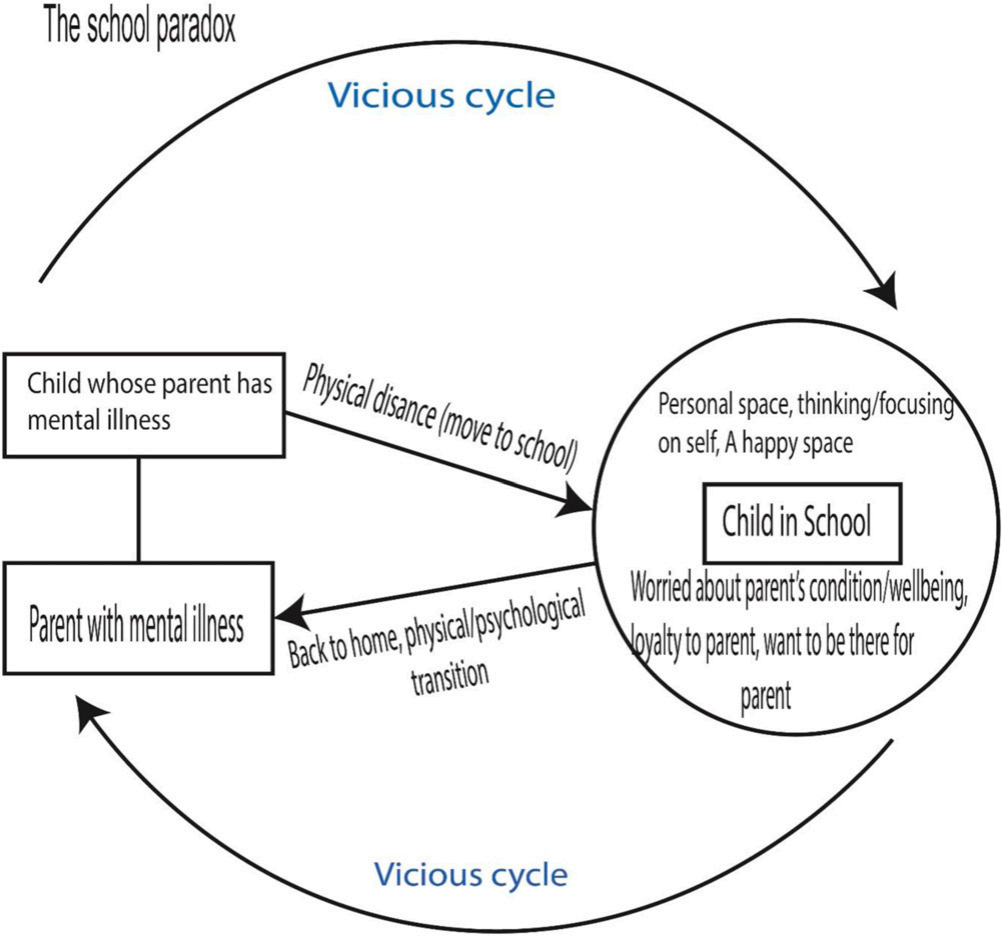
The school paradox.

The school is one of the settings that children usually frequent. Therefore, it is important to explore how having a parent with mental illness could shape their experiences at school. For many of these children, transition from the home to school is originally positive and exciting. The findings corroborate with international literature suggesting that the school can be considered a space for escape and reflection where children focus on their own lives without having to think about parental mental illness (Gladstone et al., 2011). However, children’s loyalty and responsibility to the parent with mental illness can distort the state of happiness they may enjoy once they move into the school setting. This explains the school paradox that children must confront in the context of parental mental illness (see Figure 4). Most studies about children’s interaction in the school do not clearly show the paradox. What they have often investigated is how teachers and other professionals can support the children to ensure their academic work is not impacted by parental mental illness (Bruland et al., 2017; Laletas et al., 2017). While these investigations are relevant, they do not provide insights into the problematic mechanisms that go on in the children’s lifeworld to create poor school outcomes. The construction of the school paradox enables a better understanding of a vicious cycle that develops when a child whose parent has mental illness moves to the school.

Studies of children living with parental mental illness found that they tend to enjoy a personal space where they are not being impacted by mental illness (Trondsen, 2012). In this study, the school is considered as one of the settings that produces the physical distance to enable children not to think about their parent’s mental illness. The children enjoy being at the school because it is a more structured environment, they are able to play with their friends and have fun. Living with a parent with mental illness can be physically and emotionally demanding, so children would like to create a physical and emotional distance from the parent (Fjone et al., 2009; Mordoch & Hall, 2008). However, as indicative from the study’s findings, the cycle does not end when a physical and emotional distance is created. Indeed, a physical distance is created once the child moves from the home to school. However, for many of these children, the thoughts of the parents’ condition or wellbeing continue to linger while they are in school. In fact, for some of these children they are drawn back from school to be in contact with the parent especially when the parent’s symptoms become severe and require support to be hospitalised. There were instances where some children mentioned having to leave school to attend to their parents’ needs. Therefore, even the physical distance that was originally encouraged (Fjone et al., 2009; Trondsen, 2012) loses its utility because the child eventually gets back in contact with the parent.

Meanwhile, the child’s movement back to the home from school is not always the case. This only has potential to occur if the child is significantly involved in caregiving, especially in families where there is limited social support or the other partner is unsupportive. Yet, the potential for a psychological movement back home seems unavoidable for many of these children. While the child may be physically present in school, their minds move to the home thinking about the parent’s condition. The school paradox appears inescapable for the children because they are confronted with events that seem beyond their control. This study finds that emotional and physical distance created as a result of the child moving away from the parent is not as straightforward as it seems and unless relevant support is provided to deal with the paradox, it will probably remain elusive. Critical thoughts need to be given to the children’s lifeworld to ensure that some of these challenges are not taken for granted.

### Clinical Implications

Part of the reason for the school paradox is that the school is not aware of the parent’s mental illness so teachers and other relevant professionals are unable to intervene. These children are often neglected by mental health and social services (Gray et al., 2008). It is important that, at point where the patient contacts mental health services, professionals find out whether they have children. Unfortunately, current practices suggest that mental health professionals rarely collect information about the parenting status of their patients (Campbell & Poon, 2020; Reupert et al., 2022). If mental health professionals do not find out whether their patients are parents, it would be difficult to reach these children. When mental health professionals find out that their patients have children in their care, they can relay the information to child welfare services who can then coordinate services and supports with teachers and the mental health professional. Once they are informed about the children, the social worker can contact the child’s school and assess their involvement in school activities as well as their academic performance. The social worker should work together with the teacher to ensure that negative impact on the child’s school is reduced, or even prevented. There is the possibility that teachers may have limited knowledge or not trained to respond to the needs of these children (Laletas et al., 2017; 2020). Therefore, the social worker should share non-stigmatising information about the parent’s mental illness with the teacher, the child and peers they relate with. It may also be beneficial to introduce mental health literacy programmes in schools so that peers in general are more understanding when they get to know that their colleagues have a parent with mental illness.

The school paradox also occurs because the children become worried about their parents’ wellbeing/condition. Children’s anxieties can be reduced when they know that professionals are available to provide supports. It is important that social workers and mental health professionals assure the children that they will follow-up on the parent through home visits to check up on them so that the child is able to focus on school.

### Study Limitations

The study is limited in terms of its analysis due to the depersonalisation of data as part of transcendental phenomenology. Essentially, the study did not consider demographic information or context-specific details like the age of child or school level in the analysis. This is because the focus of analysis was on the phenomenon, not the person. Therefore, some specific information in the analysis related to participants’ demographic features may be lost.

## Conclusion

The study suggests the need for multi-disciplinary collaboration to deal with the school paradox. Psychologists, mental health professionals, social workers and teachers should be involved to ensure that the child’s school involvement is not negatively impacted by parental mental illness. It is important that there is open communication among the professionals about the best ways to support these children. Breaking the school paradox involves providing assurance and hope to the child about the parent’s situation. Overall, the children’s view of the school within the context of living with a parent with mental illness provides professionals with useful suggestions to drive supports and interventions.
